# Validation of a Diabetes Subtype Classification Model Using Data from U.S. Adults Before and After the COVID-19 Pandemic

**DOI:** 10.3390/metabo16030204

**Published:** 2026-03-19

**Authors:** Brian Lu, Peng Li, Andrew B. Crouse, Tiffany Grimes, Ava N. Smith, Matthew Might, Fernando Ovalle, Anath Shalev

**Affiliations:** 1 Comprehensive Diabetes Center, Department of Medicine, Division of Endocrinology, Diabetes and Metabolism, University of Alabama at Birmingham, 1825 University Blvd, SHELBY Bldg 1272, Birmingham, AL 35233-1913, USA; 2 School of Nursing, University of Alabama at Birmingham, Birmingham, AL 35233-1815, USA; 3 Hugh Kaul Precision Medicine Institute, University of Alabama at Birmingham, Birmingham, AL 35233-2032, USA

**Keywords:** diabetes, diabetes subtype, COVID-19, pandemic (COVID-19), epidemiology

## Abstract

Background: We (and others) have previously identified five clinically distinct diabetes subtypes. Currently, few models to identify diabetes subtypes are readily accessible. Further, while COVID-19 has been associated with increased risk of new-onset diabetes, it remains unknown whether the pandemic is also associated with changes in diabetes subtype distribution. Methods: We used the electronic health records of patients diagnosed with diabetes from 2010 to 2019 at the Kirklin Clinic of the University of Alabama at Birmingham (UAB) to train models to assign diabetes subtypes previously identified by hierarchical clustering. We then applied the trained model to conduct a retrospective cluster analysis of electronic health records of patients diagnosed with diabetes from 2020 to 2024 at UAB. We further validated our findings using data from the 2015–2023 National Health and Nutrition Examination Surveys (NHANES). Results: The trained classification model had an average specificity of 98% and an average sensitivity of 93%. Using the model, we identified a significant difference in the distribution of type 2 diabetes subtypes in patients at UAB and in participants in NHANES. In particular, the proportion of patients with severe insulin-dependent diabetes or severe insulin-resistant diabetes subtypes increased from 42% to 61% and 31% to 40% at the UAB and in NHANES, respectively. Conclusions: The model presented here can facilitate the identification of diabetes subtypes. The proportions of patients with severe subtypes of diabetes have seemed to increase in the more recent years following the pandemic. Further studies are required to determine the potential causes of this phenomenon.

## 1. Introduction

Diabetes has been shown to cluster into five clinically distinct subtypes: severe autoimmune diabetes (SAID), severe insulin-deficient diabetes (SIDD), severe insulin-resistant diabetes (SIRD), mild obesity-related diabetes (MOD), and mild age-related diabetes (MARD) [1,2,3]. Corresponding to their different clinical characterizations, each subtype also has different risks of secondary complications. In particular, the severe diabetes subtypes SIDD and SIRD are associated with higher risks of cardiovascular complications and chronic kidney disease [1,2,4,5]. In contrast, the mild diabetes subtypes, MOD and MARD, come with lower risks of diabetic neuropathy [2,4,5]. As such, identifying these subtypes will allow for adjustments in treatment strategies and subsequent improvements in diabetes control. However, despite the increasing recognition of these subtypes in research and the potential for improved diabetes control, adoption of diabetes subtypes in the clinic has been slow. Furthermore, there is a lack of readily available tools to identify a patient’s particular diabetes subtype.

Diabetes has emerged as one of the comorbidities of coronavirus disease-2019 (COVID-19) [6,7]. Additionally, while it is primarily a respiratory disease caused by the severe acute respiratory syndrome-coronavirus-2 (SARS-CoV-2), COVID-19 has also been associated with increased risks of new-onset diabetes [8,9,10]. However, it remains unknown whether the pandemic has also been associated with changes in diabetes subtype distribution.

In the current study, we trained a multiclass random forest classification model to identify diabetes subtypes based on previously established subtype parameters. We then applied the model in a cross-sectional study and evaluated diabetes subtype distributions before and after the COVID-19 pandemic among two distinct cohorts of U.S. adults.

## 2. Materials and Methods

### 2.1. University of Alabama at Birmingham Cohort

We conducted a retrospective analysis of the electronic health records of adult patients with diabetes at the Kirklin Clinic of the University of Alabama at Birmingham (UAB). All human studies were approved by the UAB Institutional Review Board. Informed consent for participation was not required, as the UAB Institutional Review Board granted a waiver of HIPAA authorization. Patients with diabetes between 2010 and 2019 were selected and assigned diabetes subtypes as previously described [2]. Briefly, we screened the electronic health records of 89,875 patients diagnosed with diabetes between 1 January 2010 and 31 December 2019. Adult patients with International Classification of Diseases (ICD) diabetes codes were selected based on the availability of data for 6 established clustering parameters (GAD autoantibody, HbA1c, BMI, diagnosis age, HOMA-B, and HOMA-IR), resulting in 1194 patients being selected for subtype assignment by hierarchical clustering (Figure S1). For patients with diabetes between 2020 and 2024, 51,858 patients diagnosed with diabetes between 1 January 2020 and 23 May 2024 at the Kirklin Clinic of UAB were similarly screened and selected, resulting in 165 patients being selected for subtype assignment (Figure S2). Identical International Classification of Diseases and Logical Observation Identifiers Names and Codes (LOINC) codes were used for diagnoses and laboratory results, respectively. Diabetes subtypes for the 165 patients from 2020 to 2024 were identified using the diabetes subtype model described below. Due to the focus on type 2 diabetes, patients with severe autoimmune diabetes (SAID) were excluded, resulting in 1084 patients from 2010 to 2019 and 155 patients from 2020 to 2024 being selected for analysis.

### 2.2. National Health and Nutrition Examination Surveys Cohort

To validate our findings, we repeated the above analysis using National Health and Nutrition Examination Surveys (NHANES) data from the 2015–2023 cycles with minor modifications. Data were obtained from the 2015–2016, 2017–2020, and 2021–2023 Continuous National Health and Nutrition Examination Surveys (NHANES, unweighted *n* = 37,464) using nhanesA [11]. NHANES cycles prior to 2015 were not used because they included different methodologies for insulin and glucose measurements. Adult participants with medically diagnosed diabetes and adult participants with HbA1c ≥ 6.5% or fasting plasma glucose ≥ 7 mmol/L from the 2015–2016, 2017–2020, and 2021–2023 cycles and with available values for HbA1c, BMI, age, HOMA-B, and HOMA-IR (unweighted *n* = 1709) were selected (Figure S3). Diabetes subtypes were identified using the diabetes subtype model described below and de novo hierarchical clustering as previously described, with minor modifications [2]. GAD autoantibody measurements were not available in NHANES and were imputed as negative. The lack of GAD autoantibody measurements in NHANES precluded the identification of the SAID subtype. However, since SAID generally only accounts for a very small percentage of diabetes diagnoses [1,2], and largely represents type 1 diabetes, it was not relevant for this type 2 diabetes-focused analysis. Also, as c-peptide values were not available, and insulin-derived HOMA1 estimates are not directly interchangeable with c-peptide-derived HOMA1 estimates, insulin values were used for HOMA-B and HOMA-IR calculations then scaled using the HOMA1 mixed-effect model described below. Age at time of survey was used instead of age at time of initial diabetes diagnosis due to the large time gap between the initial diabetes diagnosis and the survey and is a limitation that may affect subtype assignment. However, as a person’s diabetes subtype may change over time [12], age at time of survey, when the other clinical parameters were measured, should provide a better reflection of the participant’s diabetes subtype at the time of the survey. Participants with zero adjusted weights were removed, resulting in 1132 participants from 2015 to 2020 and 470 participants from 2021 to 2023 being selected for analysis.

### 2.3. Diabetes Subtype Classification Model

To identify diabetes subtypes, we trained and evaluated multiple multiclass classification models using UAB patients with diabetes from 2010 to 2019 with diabetes subtypes previously identified using hierarchical clustering [2]. Model subtype assignment was based on the 6 clustering parameters described above (Supplemental Methods). Models were trained using the scikit-learn Python machine learning library version 1.5.2 with 80% of the UAB patients from 2010 to 2019 for training and 20% of the UAB patients from 2010 to 2019 for testing. The optimal hyper-parameters were selected using grid search with 5-fold rotation estimation repeated 5 times. The final model was chosen based on average rotation estimation performance metrics during training. Final model metrics were evaluated on the holdout 20% testing set of UAB patients from 2010 to 2019.

### 2.4. HOMA1 Mixed-Effect Model

As the HOMA1_c-peptide_ values we used for classification were not directly comparable to HOMA1_insulin_ values, we constructed a linear mixed-effect model to scale HOMA1_insulin_ values to comparable HOMA1_c-peptide_ values. HOMA1_insulin_ values were calculated as originally described by Matthews et al. [13]. The mixed-effect model was constructed using records from UAB (*n* = 2720) and from the 1999–2004 NHANES cycles (unweighted *n* = 6331) where patients/survey participants had their insulin, c-peptide, and glucose measured simultaneously (3003 and 6510 HOMA1_insulin_/HOMA1_c-peptide_ pairs from UAB and NHANES, respectively). Data from 80% of the patients/survey participants were used for training and data from 20% of the patients/survey participants were used for testing. Self-reported sex and race were included as fixed effects, as both have previously been reported to contribute to differences in insulin clearance rates [14,15]. The model included random intercepts for patients/survey participants to account for repeated measures. To obtain the population average, only fixed effects were used to scale HOMA1_insulin_ values to HOMA1_c-peptide_ values.

### 2.5. Statistical Analysis

Statistical analyses were performed using the R software version 4.4.0 (R Foundation). Differences between continuous parameters were analyzed using Student’s *t* test. Differences in categorical variables were analyzed using Pearson’s Chi-squared test. The association between time periods and diabetes severity within each cohort were also assessed using multivariable logistic regression to adjust for sex, race, age, BMI, and diabetes diagnosis status as appropriate. NHANES data was analyzed with adjusted fasting subsample 2 year mobile exam center (MEC) weights according to the NHANES analytic guidelines to account for the complex survey design. Confidence intervals for area under the receiver operator characteristics curves were generated by bootstrapping.

### 2.6. Code Availability

Our diabetes subtype classification application, DiaClue, is accessible at https://diaclue.com/ (accessed on 12 March 2026). The code for DiaClue is available at https://github.com/avasmith98/diabetes-cluster-app (accessed on 23 February 2026). The codes for model training and data analysis are available at https://github.com/brianluhd/cluster_prediction (accessed on 13 November 2025) and https://github.com/brianluhd/COVID19_clusters (accessed on 29 January 2026), respectively.

## 3. Results

**Figure 1:**
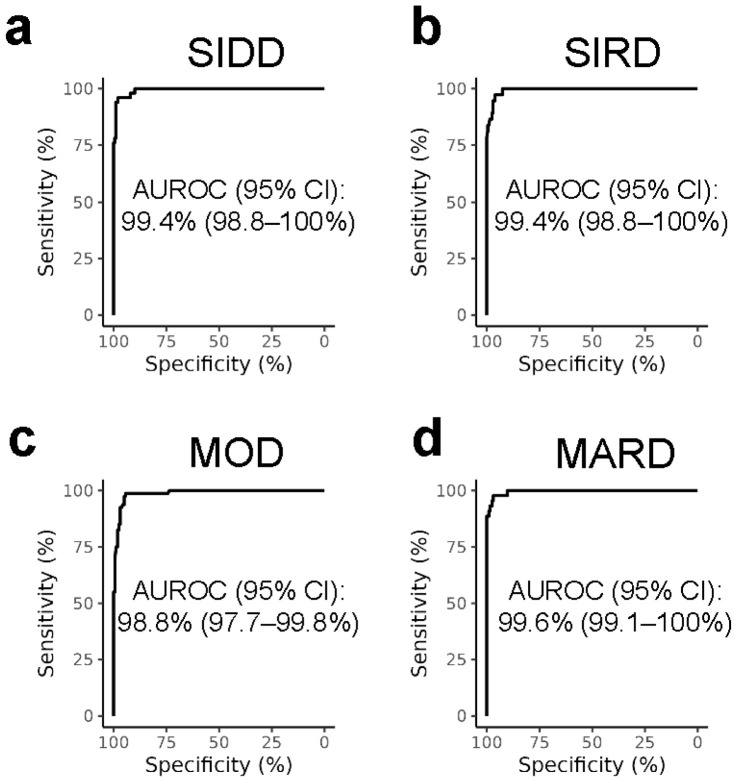
Area under the receiver operator characteristics curve for severe insulin-deficient diabetes (SIDD) vs. others (**a**), severe insulin-resistant diabetes (SIRD) vs. others (**b**), mild obesity-related diabetes (MOD) vs. others (**c**), and mild age-related diabetes (MARD) vs. others (**d**). Area under the receiver operator characteristics (AUROC) for severe autoimmune diabetes (SAID) vs. others, based on the binary GAD autoantibody positivity, is 100% and is not shown.

To assign diabetes subtypes, we trained multiclass random forest, gradient boosting, histogram-based gradient boosting, and Gaussian Naive Bayes classification models using UAB patients with previously assigned diabetes subtypes from 2010 to 2019 [2]. All models, besides the Gaussian Naive Bayes models, achieved similar performances (Table S1). Model performances were comparable when trained on either the non-linear HOMA2 values or the linear approximation HOMA1_c-peptide_ values, but slightly worse when trained on glucose and c-peptide values directly (Table S1). The random forest model using HOMA1_c-peptide_ values was chosen for its simplicity and interpretability and comparable performance to the more complex, but less interpretable, models. The final random forest model had an average specificity of 98% and an average sensitivity of 93%. The area under the receiver operator characteristics curves were over 98% for each diabetes subtype (Figure 1).

As our HOMA1_c-peptide_ values used for classification were not directly comparable to HOMA1_insulin_ values, direct use of HOMA1_insulin_ values in the trained random forest model resulted in weak agreement with subtypes previously assigned by hierarchical clustering using the c-peptide-derived non-linear HOMA2 [2] (Table S2). Therefore, to allow the use of our diabetes subtype classification model on NHANES data with HOMA1_insulin_ values, we also constructed a linear mixed-effect model using HOMA1_insulin_ and HOMA1_c-peptide_ estimate pairs from UAB and NHANES (Figure S4). Diabetes subtype classification with the random forest model using scaled HOMA1_insulin_ showed strong agreement with subtypes previously assigned by hierarchical clustering [2] (Table S2), indicating that our mixed-effect model can effectively scale insulin-derived HOMA values for downstream diabetes subtype classification without having a substantial impact on model performance.

Using the trained random forest model, we assigned diabetes subtypes to patients with diabetes from UAB between 2020 and 2024. Interestingly, the distribution of diabetes subtypes was significantly different between 2010–2019 and 2020–2024 in the UAB cohort (*p* < 0.001), while cohort demographics did not change overall, and the proportions of males and Black/African Americans remained similar (Table 1). In particular, the proportion of patients with severe subtypes of diabetes (severe insulin-deficient diabetes and severe insulin-resistant diabetes) increased from 42% (455/1084) in 2010–2019 to 61% (94/155) in 2020–2024, while the proportion of patients with mild subtypes of diabetes (mild obesity-related diabetes and mild age-related diabetes) decreased from 58% (629/1084) to 39% (61/155) (*p* < 0.001). The increase in severe diabetes subtypes remained significant even after adjusting for sex, race, age, and BMI (adjusted odds ratio [AOR], 1.96; 95% CI 1.38–2.79; *p* < 0.001).

To validate our findings, we repeated the analysis using the 2015–2023 NHANES data (Table 2). The diabetes subtypes assigned by the trained random forest model were similar to those assigned by de novo hierarchical clustering (Figure S5). While NHANES had also similar proportions of males and Black/African American participants between 2015–2020 and 2021–2023, there was again a significant difference between the distribution of diabetes subtypes (*p* = 0.047). The proportion of survey participants with severe subtypes of diabetes increased from 31% (weighted, 354/1132) in 2015–2020 to 40% (weighted, 172/470) in 2021–2023, while the proportion of participants with mild subtypes of diabetes decreased from 69% (weighted, 778/1132) to 60% (weighted, 298/470) (*p* = 0.006). The increase in severe diabetes subtypes remained similarly significant after adjusting for sex, race, age, and BMI (AOR, 1.45; 95% CI, 1.08–1.95; *p* = 0.015). Similar increases in severe diabetes subtypes were again observed in NHANES using subtypes assigned by de novo hierarchical clustering (Table S3). Surprisingly, only a small fraction of people assigned the SIDD subtype were prescribed insulin in this cohort (weighted 27%, 35/98).

## 4. Discussion

In the current study, we trained a multiclass random forest classification model to identify diabetes subtypes using established clustering parameters. Using this model, our studies identified an increase in the proportions of patients with severe subtypes of diabetes in the more recent years following the pandemic. We also observed a surprisingly low rate of insulin usage in patients with SIDD in the NHANES cohort, suggesting that severe insulin deficiency in patients with diabetes may not be adequately recognized and managed and underlining the need for tools to facilitate better recognition and management. Since severe subtypes of diabetes have an increased lifetime risk of diabetes complications [1,2,4,5], recognizing and adequately adjusting treatment of these diabetes subtypes should help improve the outcome.

The limitations of this study include the relatively low number of patients with diabetes and complete data in the UAB and NHANES cohorts. Our analysis was therefore also limited to the time periods before and after the COVID-19 pandemic instead of a more granular time scale. However, our findings were consistent across the two cohorts, and the weight adjustments methods applied to NHANES data produced nationally representative estimates. Our findings are also consistent with the increase in HbA_1c_ and decrease in glycemic control that were recently found in NHANES [16].

While HOMA estimates for the NHANES cohort were derived from fasting glucose, as it was part of the survey design, the glucose values used to calculate HOMA estimates for the UAB cohort were presumed to be non-fasting, as the fasting status was not specified in the UAB electronic health records, and this could theoretically affect classification. Nonetheless, our findings were still consistent across the two cohorts, which is in alignment with previous findings that fasting and non-fasting HOMA estimates are similar and correlate [17].

Also, the established diabetes classification model used in the present studies does not take into account the use of any glucose-lowering medications, which may affect subtype assignment and confound the underlying diabetes etiology.

With the current studies being observational, further studies are required to determine the causes of the increase in severe diabetes subtypes, but potential explanations may include direct biological effects from SARS-CoV-2 infection and indirect effects from the pandemic lockdowns and the resulting disruption of diabetes services. COVID-19 is associated with increased risk of new-onset diabetes [6,7], and SARS-CoV-2 has been shown to be able to directly infect pancreatic beta cells, impair beta cell function, and induce beta cell apoptosis [18]. The associated inflammation from SARS-CoV-2 infection can also induce beta cell pyroptosis [19]. Furthermore, although BMI remained unchanged in our study cohorts, SARS-CoV-2 can increase insulin resistance through direct infection of adipocytes [20]. Potential disruptions in health services and diabetes care during the pandemic include limited access to routine care and medications, increased use of telemedicine, and changes in patient care-seeking behavior [21]. Pandemic-associated disruptions in diabetes services may have also led to a delay in diabetes diagnosis, allowing diabetes to worsen prior to diagnosis. However, recent estimates did not identify any changes in the age-adjusted prevalence of total, diagnosed, and undiagnosed diabetes following the COVID-19 pandemic [16,22], making this possibility unlikely.

Previous studies have also studied the correlation between insulin-derived HOMA and c-peptide-derived HOMA values [23,24], and while it was not significant in people from Cameroon without diabetes [24], a significant correlation between insulin-derived HOMA-IR and c-peptide-derived HOMA-IR was found in people from India who have diabetes [23], which was consistent with our findings.

Although the diabetes subtypes initially described by Ahlqvist et al. have been well replicated across multiple populations and are generally similar across populations [25,26,27], cross-validation of clusters has shown varying results when cluster centers from one cohort were applied to other cohorts [27]. As such, while we were able to successfully apply our diabetes subtype classification model trained on the UAB cohort to the nationally representative NHANES cohort, no independent clinical validation was possible, and the performance of our model on populations outside of the United States that were not represented in the training cohort remains unknown and may be limited.

To facilitate the use of our model to identify subtypes of adult-onset, non-monogenic, non-gestational diabetes, we created DiaClue, which can be accessed as a web application and as a downloadable application on mobile devices. Users can directly input the six clinical parameters required for cluster assignment and DiaClue will output the most likely subtype assignment. Recognizing that a person with diabetes may have clinical features that overlap with multiple subtypes, DiaClue also outputs a percentage score for each subtype, along with a score breakdown using Shapley additive explanations for the most likely subtype [28], to aid users in making their independent and balanced subtype assignment. A few studies have previously described diabetes subtype classification models [12,29,30,31,32,33]. To our knowledge, no publicly accessible model trained on a recent U.S. cohort was previously available. The model described by Bello-Chavolla et al. was trained on NHANES III data from 1988 to 1994 [12], the model described by Mori et al. [31] used the All-New Diabetes in Scania (ANDIS) cluster centroids originally identified by Ahlqvist et al. [1], and the model described by Baskar et al. was trained on a cohort from India [33]. In comparison, our model was trained on the more recent 2010–2019 data from a multiracial patient cohort from the Kirklin Clinic at UAB, a large academic multispecialty clinic in the US Deep South [2].

The impact of identifying diabetes subtypes on treatment and outcome using our application and in general has yet to be evaluated in prospective, randomized clinical trials. However, our application can provide information relevant to pathophysiology and complication risks and thereby support clinicians in their tailoring of diabetes management strategies. Although current clinical guidelines do not include diabetes subtypes, strategies for subtype-specific management have been proposed [34,35,36]. A summary of these therapeutic considerations for subtype-specific diabetes management strategies according to prior reviews is provided in Table 3.

## 5. Conclusions

The model presented here can facilitate the identification of diabetes subtypes. Interestingly, we found that the proportions of patients with severe subtypes of diabetes increased in the more recent years following the pandemic. While further studies are required to examine the potential causes of this phenomenon, diagnosing these diabetes subtypes and identifying trends early will support better tailoring of diabetes management strategies and should improve diabetes control and outcomes.

## Figures and Tables

**Table 1:** Characteristics and subtype distribution of adult patients with diabetes at the University of Alabama in Birmingham.

Period	2010–2019 (*n* = 1084)	2020–2024 (*n* = 155)	*p*
Sex, No. (%)			
Male	445 (41)	70 (45)	0.377
Female	639 (59)	85 (55)	
Race, No. (%)			
White	557 (51)	76 (49)	0.165
AA	454 (42)	62 (40)	
Other	73 (7)	17 (11)	
Age, mean (95% CI), years	54.9 (54.2–55.7)	48.1 (45.7–50.5)	<0.001
BMI, mean (95% CI), kg/m^2^	33.2 (32.8–33.6)	33.1 (31.9–34.2)	0.876
Diabetes subtype, No. (%)			
SIDD	285 (26)	54 (35)	<0.001
SIRD	170 (16)	40 (26)	
MOD	380 (35)	41 (26)	
MARD	249 (23)	20 (13)	
Diabetes severity, No. (%)			
Severe (SIDD and SIRD)	455 (42)	94 (61)	<0.001
Mild (MOD and MARD)	629 (58)	61 (39)	

Abbreviations: AA—Black or African American; SIDD—severe insulin-deficient diabetes; SIRD—severe insulin-resistant diabetes; MOD—mild obesity-related diabetes; MARD—mild age-related diabetes.

**Table 2:** Characteristics and subtype distribution of U.S. adults with diabetes.

Period	2015–2020 (*n* = 1132)	2021–2023 (*n* = 470)	*p*
Sex, No. (%) ^a^			
Male	585 (53)	242 (56)	0.463
Female	547 (47)	228 (44)	
Race, No. (%) ^a^			
White	325 (58)	253 (54)	0.672
AA	274 (13)	62 (16)	
Other	533 (29)	155 (30)	
Age, mean (95% CI) ^a^, years	59.5 (58.5–60.5)	60.2 (58.5–61.9)	0.494
BMI, mean (95% CI) ^a^, kg/m^2^	32.9 (32.3–33.6)	33.6 (32.9–34.2)	0.173
Diabetes subtype, No. (%) ^a^			
SIDD	85 (7)	32 (11)	0.047
SIRD	269 (24)	140 (29)	
MOD	336 (31)	118 (28)	
MARD	442 (38)	180 (32)	
Diabetes severity, No. (%) ^a^			
Severe (SIDD and SIRD)	354 (31)	172 (40)	0.006
Mild (MOD and MARD)	778 (69)	298 (60)	

Abbreviations: AA—Non-Hispanic Black American; SIDD—severe insulin-deficient diabetes; SIRD—severe insulin-resistant diabetes; MOD—mild obesity-related diabetes; MARD—mild age-related diabetes. ^a^ Nationally representative estimates after weighting according to the NHANES analytic guidelines.

**Table 3:** Diabetes subtype therapeutic considerations.

Diabetes Subtype	Subtype Characteristics	Risks/Complications	Therapeutic Considerations
SAID	GAD autoantibody-positive	Microvascular complications [5,35] DKA [1,35,36]	For most individuals with T1D, early and aggressive insulin replacement is recommended, and the use of SGLT2 inhibitors is potentially unsafe [36,37]. For individuals with LADA, a subset of T1D [38], approved guidelines for T1D or modified guidelines for type 2 diabetes may be considered depending on the individual’s remaining beta cell function [38].
SIDD	High HbA1c Insulin deficiency as indicated by low c-peptide GAD autoantibody-negative	Micro- and macrovascular complications [2,5,26,35,39] DKA [1,35,36]	Aggressive glucose control is recommended [36]. Insulin secretagogues, including incretin-based therapies, could be considered [34,36], and early treatment with insulin might be necessary and beneficial [26,34,36,37]. SGLT2 inhibitors may also be considered in those with heart or kidney disease, but their use should be carefully weighed against the risk of diabetic ketoacidosis and closely monitored [37].
SIRD	Insulin resistance as indicated by high c-peptide and glucose GAD autoantibody-negative	Nephropathy [5,26,35,36,39] MAFLD [26,35,36] CVD [5,26,35,36,39]	Aggressive glucose control is recommended [36]. Early treatment with SGLT2 inhibitors or GLP1 RAs as well as adjuvant therapy with metformin could be considered [34,35,36]. Insulin may be considered later in the disease process to help achieve better glycemic control [34].
MOD	Relatively high body mass index GAD autoantibody-negative	Lower complication risk [2,4]	Weight loss with diet and exercise could be considered [34,35]. Metformin, SGLT2 inhibitors, and GLP1 RAs might be beneficial as first-line pharmacological therapies [34,35].
MARD	Higher age at diagnosis GAD autoantibody-negative	Lower complication risk [2,4]	A more conservative therapeutic approach with lifestyle modifications and medications with low risk of hypoglycemia might be appropriate [34,35,40].

Abbreviations: SAID—severe autoimmune diabetes; SIDD—severe insulin-deficient diabetes; SIRD—severe insulin-resistant diabetes; MOD—mild obesity-related diabetes; MARD—mild age-related diabetes; GAD—glutamate decarboxylase; HbA1c—hemoglobin A1c; DKA—diabetic ketoacidosis; MAFLD—metabolic associated fatty liver disease; CVD—cardiovascular disease; T1D—type 1 diabetes; SGLT2—sodium-glucose cotransporter 2; LADA—latent autoimmune diabetes in adults; GLP1 RAs—glucagon-like peptide-1 receptor agonists.

## Data Availability

Restrictions apply to the availability of the UAB data generated or analyzed during this study to preserve patient confidentiality or because they were used under license. The corresponding author will, on request, detail the restrictions and any conditions under which access to some data may be provided. NHANES data is publicly available at https://www.cdc.gov/nchs/nhanes/index.html, accessed on 12 March 2026.

## Supplementary Materials

The following supporting information can be downloaded at https://www.mdpi.com/article/10.3390/metabo16030204/s1, Supplemental Methods: Additional details on HOMA calculations, Table S1: Diabetes subtype assignment model selection, Table S2: Model performance of subtype assignment against subtypes previously assigned from hierarchical clustering with c-peptide-derived HOMA2 in UAB patients, Table S3: Subtype distribution of U.S. adults with diabetes using de novo hierarchical clustering, 2015–2023, Figure S1: Exclusion cascade for UAB patients with diabetes from 2010 to 2019, Figure S2: Exclusion cascade for UAB patients with diabetes from 2020 to 2024, Figure S3: Exclusion cascade for NHANES participants from 2015 to 2023, Figure S4: Relationship between c-peptide- and insulin-derived HOMA1-B and HOMA1-IR in the training and testing data, Figure S5: Subtype characteristics of NHANES participants with diabetes in 2015–2023, Figure S6: Average impact on the random forest model output for each parameter and diabetes subtype.
