# Supplementary material for: Validation of a Diabetes Subtype Classification Model Using Data from U.S. Adults Before and After the COVID-19 Pandemic
Source: Metabolites. 2026 Mar 19;16(3):204. doi: 10.3390/metabo16030204 (PMC13028042; doi:10.3390/metabo16030204)
Supplement: Supplementary file 1 [file metabolites-16-00204-s001.zip › metabolites-4194203-supplementary.pdf]

Supplemental Methods

Model subtype assignment was based on the 6 established clustering parameters (GAD autoantibody, HbA1c, BMI, diagnosis age, HOMA-B, and HOMA-IR). Linear approximations of homeostatic model assessment for c-peptide ($\text{HOMA1}_{\text{c-peptide}}$) was approximated by substituting insulin for c-peptide in the HOMA1 formulas as:

$$\text{HOMA1−B}_{\text{c−peptide}} = \frac{20 \cdot (\text{c−peptide})}{\text{glucose} - 3.5} \cdot 6$$

$$\text{HOMA1−IR}_{\text{c−peptide}} = \frac{\text{glucose} \cdot (\text{c−peptide})}{22.5} \cdot 6$$

Where glucose is in mmol/L, c-peptide is in nmol/L, and 6 is a scaling factor. The $\text{HOMA1}_{\text{c-peptide}}$ values, though correlated with HOMA2 values (UAB 2010-2019 cohort: $\text{HOMA1-B}_{\text{c-peptide}}$ $r = 0.916$, $p < 0.001$; $\text{HOMA1-IR}_{\text{c-peptide}}$ $r = 0.796$, $p < 0.001$), were not directly comparable to insulin-derived HOMA1 ($\text{HOMA1}_{\text{insulin}}$) or HOMA2 values. However, since tree-based machine learning models are not sensitive to feature scaling, we saw similar performances between models trained on either HOMA2 values or $\text{HOMA1}_{\text{c-peptide}}$ values (Table S1).

Table S1. Diabetes subtype assignment model selection

	Random forest			Gradient boost		Histogram-based gradient boost		Gaussian Naïve Bayes	
metric	HOMA2	HOMA1	Direct*	HOMA1	Direct*	HOMA1	Direct*	HOMA1	Direct*
accuracy	0.90965	0.917192	0.890603	0.911749	0.895279	0.923456	0.908227	0.83673	0.797394
precision	0.903096	0.907356	0.890211	0.905889	0.893919	0.916967	0.903412	0.837869	0.807055
recall	0.901152	0.905759	0.886911	0.903874	0.891518	0.914764	0.900942	0.834124	0.802078
F1	0.901171	0.905781	0.886093	0.903827	0.891062	0.914793	0.9007	0.834151	0.80193
ROC	0.988356	0.988377	0.98428	0.983255	0.984915	0.990187	0.987801	0.959817	0.950019

* c-peptide and glucose directly

Table S2. Model performance of subtype assignment against subtypes previously assigned from hierarchical clustering with c-peptide-derived HOMA2 in UAB patients

metric	insulin HOMA1	Scaled insulin HOMA1
accuracy	0.701369	0.877595
precision	0.865975	0.897436
recall	0.620482	0.873494
F1	0.633345	0.879255
Kappa	0.532227	0.836399

Table S3. Subtype distribution of U.S. adults with diabetes using *de novo* hierarchical clustering, 2015-2023

Period	2015-2020 (n = 1132)	2021-2023 (n = 470)	P
Diabetes subtype, No. (%)[a]			
SIDD	150 (10)	55 (15)	0.014
SIRD	134 (14)	86 (19)	
MOD	380 (38)	107 (27)	
MARD	468 (37)	222 (39)	
Diabetes severity, No. (%)[a]			
Severe (SIDD & SIRD)	284 (24)	141 (34)	0.005
Mild (MOD & MARD)	848 (76)	329 (66)	

The increase in severe diabetes subtypes remained significant even after adjusting for sex, race, age, BMI, and diabetes diagnosis status (adjusted odds ratio = 1.54, 95% CI, 1.09-2.17, p = 0.015.)

Abbreviations: SIDD, severe insulin-deficient diabetes; SIRD, severe insulin-resistant diabetes; MOD, mild obesity-related diabetes; MARD, mild age-related diabetes

[a] Nationally representative estimates after weighting according to the NHANES analytic guidelines

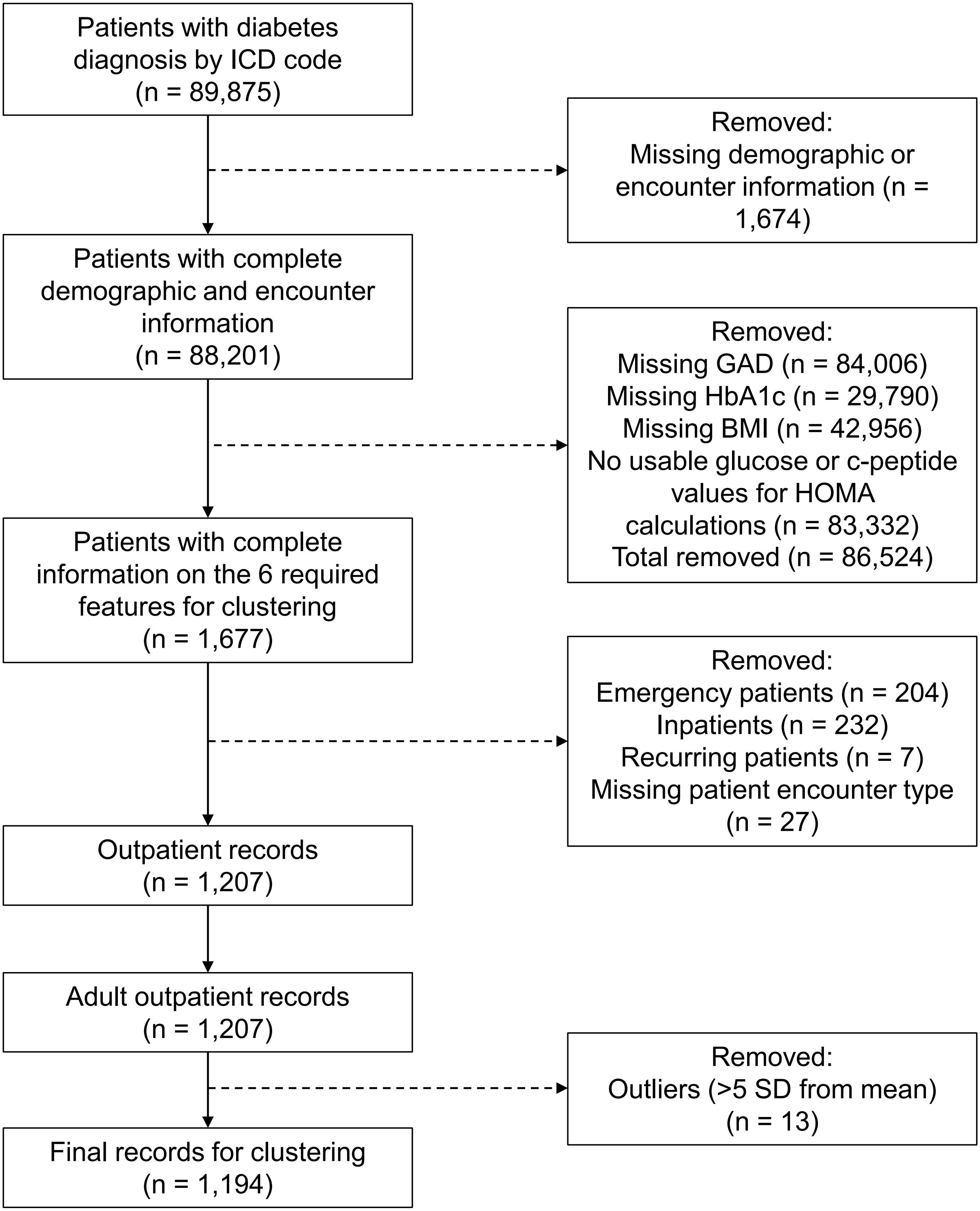

Figure S1. Exclusion cascade for UAB patients with diabetes from 2010-2019. Patients with missing data were removed, and only patients with all data available were included in the complete case analysis. The sum of patients removed for each missing parameter within a single box may not equal the total number of patients removed at that step as patients may be missing multiple parameters.

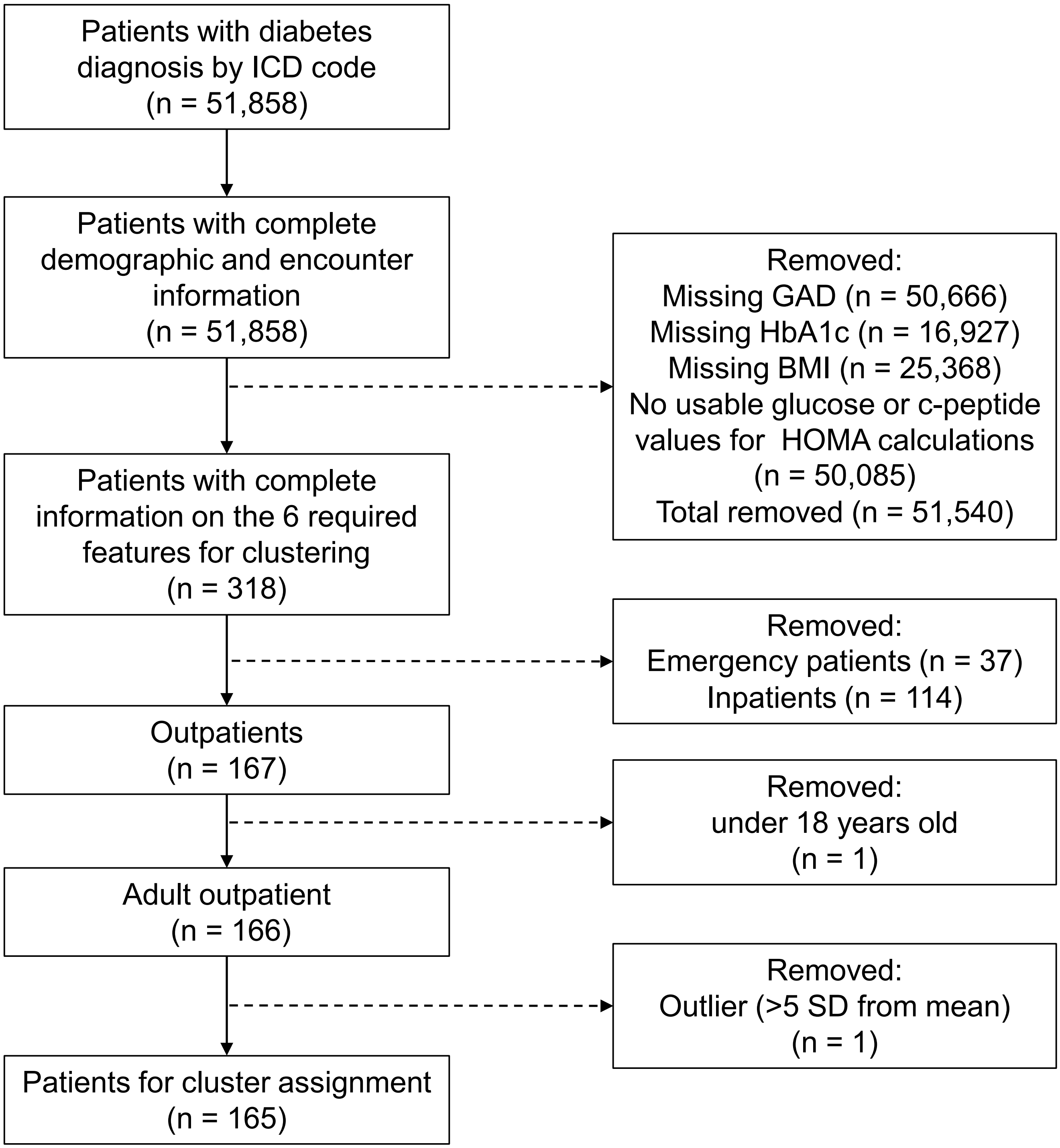

Figure S2. Exclusion cascade for UAB patients with diabetes from 2020-2024. Patients with missing data were removed, and only patients with all data available were included in the complete case analysis. The sum of patients removed for each missing parameter within a single box may not equal the total number of patients removed at that step as patients may be missing multiple parameters.

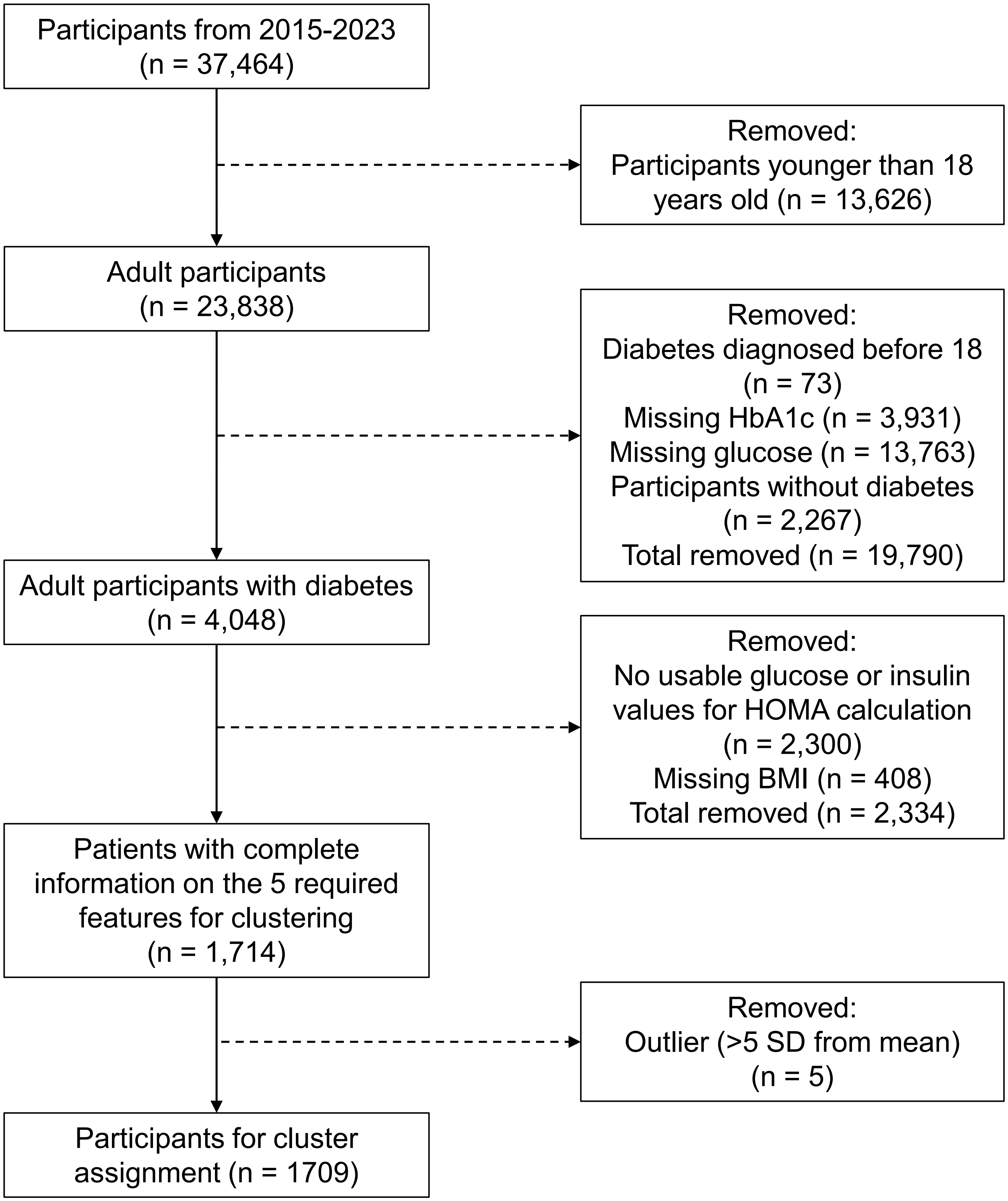

Figure S3. Exclusion cascade for NHANES participants from 2015-2023. N shown are unweighted counts. Participants with missing data were removed, and only participants with all data available were included in the complete case analysis. The sum of participants removed for each missing parameter within a single box may not equal the total number of participants removed at that step as participants may be missing multiple parameters.

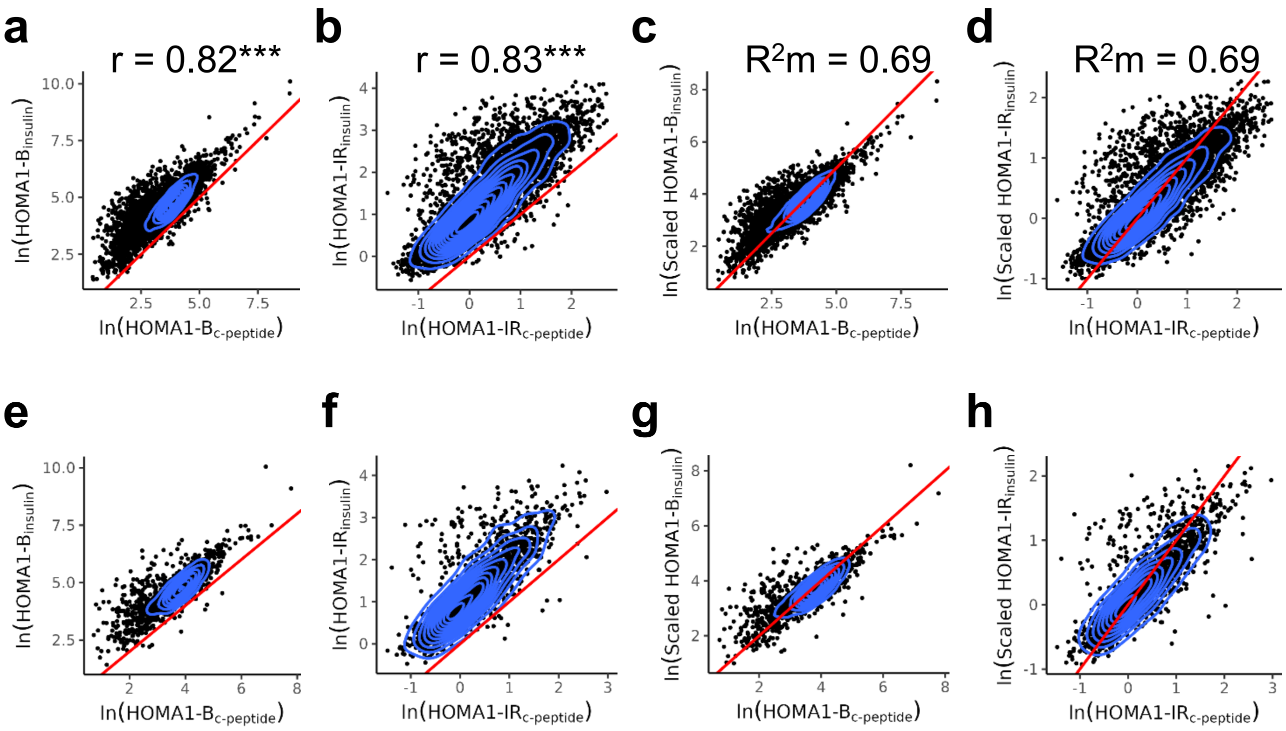

Figure S4. Relationship between c-peptide- and insulin-derived HOMA1-B and HOMA1-IR in the training (a, b) and testing (e, f) data. Natural log-transformed insulin-derived HOMA1 values showed strong correlation with natural log-transformed c-peptide-derived HOMA1 values. Observed c-peptide-derived HOMA1-B and HOMA1-IR compared to scaled insulin-derived HOMA1-B and HOMA1-IR in the training (c, d) and testing (g, h) data. The fitted linear mixed-effect models were able to explain the majority of the variance for both HOMA1-B (marginal R^2 = 0.69, conditional R^2 =0.85) and HOMA1-IR (marginal R^2 = 0.69, conditional R^2 = 0.82). Blue circles denotes density of observations. Red line denotes perfect fit. Abbreviations: r, Pearson’s product-moment correlation coefficient; R^2m, marginal R^2. *** $p < 0.001$

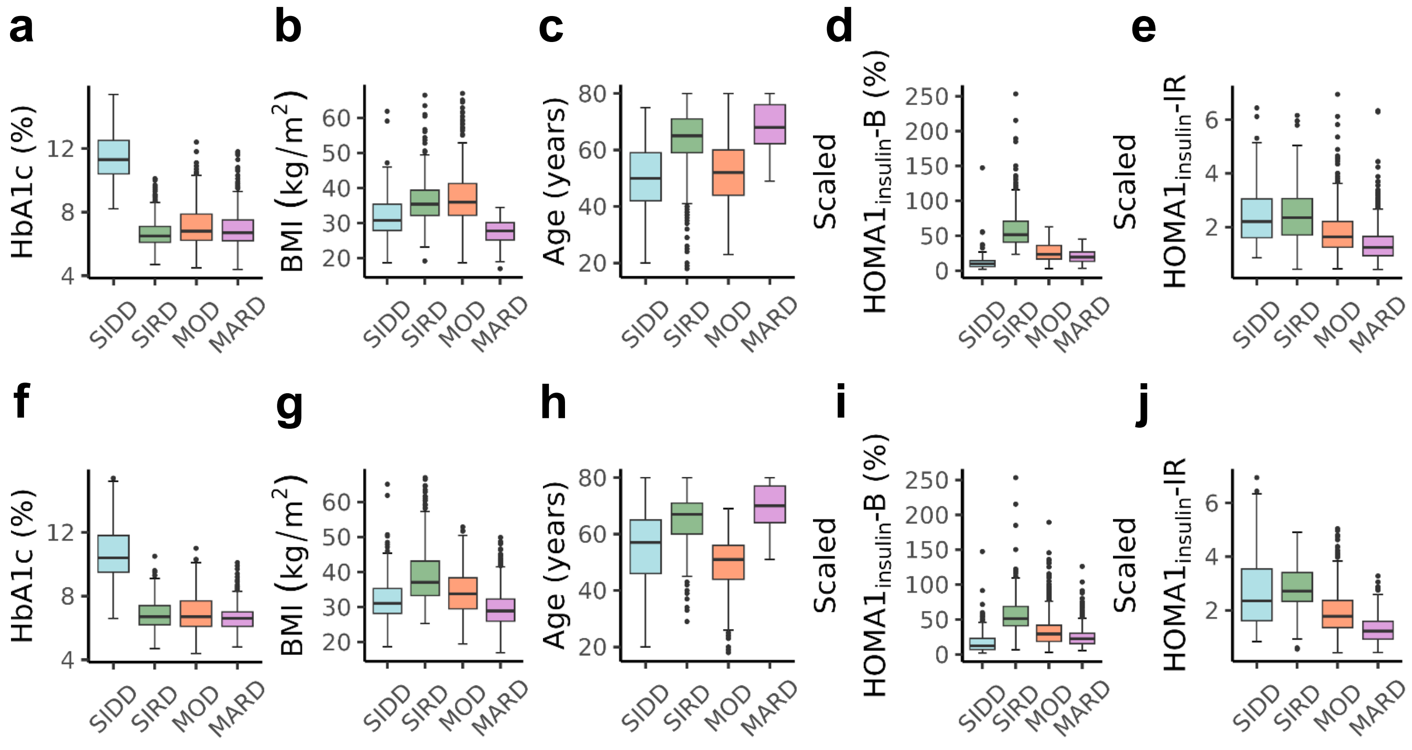

Figure S5. Subtype characteristics of NHANES participants with diabetes in 2015-2023. Subtype characteristics demonstrating the distribution of HbA1c, BMI, diagnosis age, Scaled $HOMA1_{insulin}$-B, and Scaled $HOMA1_{insulin}$-IR for each cluster assigned by the random forest model (a-e) or by *de novo* hierarchical clustering (f-j). $HOMA1_{insulin}$ values were scaled using the mixed-effect model. Points represent outliers less than or greater than 1.5 times the interquartile range from the first and third quartiles, respectively.

Abbreviations: BMI, body mass index; HOMA1-B, homoeostatic model assessment estimates of β-cell function; HOMA1-IR, homoeostatic model assessment estimates of insulin resistance; SIDD, severe insulin-deficient diabetes; SIRD, severe insulin-resistant diabetes; MOD, mild obesity-related diabetes; MARD, mild age-related diabetes

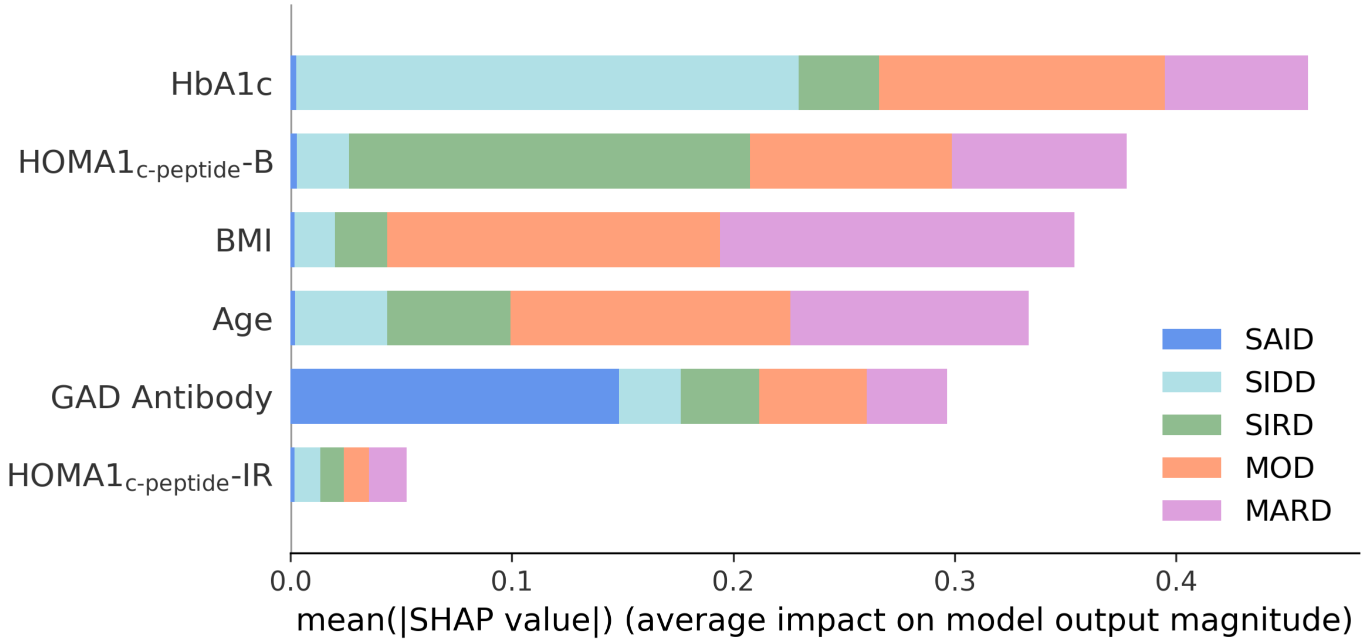

Figure S6. Average impact on the random forest model output for each parameter and diabetes subtype. Values are shown as mean absolute value of Shapley additive explanation values.

Abbreviations: HOMA1-B, homoeostatic model assessment estimates of β-cell function; BMI, body mass index; GAD, glutamate decarboxylase; HOMA1-IR, homoeostatic model assessment estimates of insulin resistance; SAID, severe autoimmune diabetes; SIDD, severe insulin-deficient diabetes; SIRD, severe insulin-resistant diabetes; MOD, mild obesity-related diabetes; MARD, mild age-related diabetes; SHAP, Shapley additive explanation
